# Maternal undernutrition and excessive body weight and risk of birth and health outcomes

**DOI:** 10.1186/s13690-017-0181-0

**Published:** 2017-02-03

**Authors:** Md Nuruzzaman Khan, Md Mizanur Rahman, Asma Ahmad Shariff, Md Mostafizur Rahman, Md Shafiur Rahman, Md Aminur Rahman

**Affiliations:** 1 Department of Population Science and Human Resource Development, University of Rajshahi, 6205 Rajshahi, Bangladesh; 2 Department of Global Health Policy, University of Tokyo, Tokyo, Japan; 3 Centre for Foundation Studies in Science, University of Malaya, Malaya, Malaysia

**Keywords:** Maternal BMI, Dual nutritional burden, Birth and health outcome, Bangladesh

## Abstract

**Background:**

Overweight and obesity are increasing in low- and middle-income countries, while underweight remains a significant health problems. However, the association between double burden of nutrition and risk of adverse birth and health outcomes is still unclear in Bangladesh. The aim of this study was to determine the effect of maternal undernutrition and excessive body weight on a range of maternal and child health outcomes.

**Methods:**

In this study, we used Bangladesh Demographic and Health Survey (BDHS) 2011 and 2014 data sets to cover the maternal, child and non-communicable diseases related health outcomes. The study considered a range of outcome variables including pregnancy complication, cesarean delivery, diabetes, hypertension, stunting, and wasting, low birth weight, genital discharge, genital sore/ulcer, stillbirth, early neonatal mortality, perinatal mortality, preterm birth and prolonged labor. The key exposure variable was maternal body mass index. Multilevel regression analysis was performed to examine the association between outcomes and exposure variables.

**Results:**

Maternal overweight and obesity has increased from 10% in 2004 to 24% in 2014, a 240% increase in 10 years. Between 2004 and 2014, maternal undernutrition declined from 33% to 18%, a reduction rate of only 54% in 10 years. Compared to normal-weight women, overweight and obese women were more likely to have experienced pregnancy complication, cesarean delivery, diabetes, and hypertension. Underweight women were 1.3 times more likely to have children with stunting and 1.6 times more likely to experience wasting compared to normal weight women. Maternal BMI was not significantly associated with increased risk of genital sore or ulcer, genital discharge, menstrual irregularities, or low birth weight though in certain cases risk was higher.

**Conclusions:**

High maternal overweight and obesity were observed to have significant adverse effects on health outcomes, while underweight was a risk factor for newborn health. The findings show that weight management is necessary to prevent adverse birth and health outcomes in Bangladesh.

**Trial registration:**

Data related to health was collected by following the guidelines of ICF international and Bangladesh Medical Research Council. The registration number of data collection is 132989.0.000 and the data-request was registered on March 11, 2015.

**Electronic supplementary material:**

The online version of this article (doi:10.1186/s13690-017-0181-0) contains supplementary material, which is available to authorized users.

## Background

Globally, overweight and obesity increasing rapidly especially in low- and lower middle- income countries. It has risen to 27.5% between 1980 and 2013 worldwide [1]. If secular trends continue, by 2030 there will be 2.16 billion overweight and 1.12 billion obese [2]. Overweight and obesity has become a major health problem both in developed and in developing countries. High-income countries reported more than double prevalence of obesity than low- and lower middle- income countries [3]. Men in developed countries were more overweight and obese, whereas in developing countries, overweight and obesity were more prevalent in women [3].

A wide variation of high BMI was observed between regions, country income categories and age-sex distribution [4]. High BMI was more prevalent in the American region (62% for overweight in both sexes, and 26% for obesity) and bit lower in the South East Asia region (14% overweight in both sexes and 3% for obesity) [5]. Asian countries have some of the lowest prevalence’s of overweight and obesity, but prevalence rates are increasingly rapidly [6, 7]. The boom in economic development and cultural factors are often cited as drivers. Almost one-half of the Indian adults living in urban area are overweight and obese [8]. Similar nutritional shifts were also observed during the last few decades in Asian countries including Pakistan [9], Nepal [10], and Bangladesh [11]. This may incur a high burden of nutrition-related diseases in these countries.

The growing epidemic of maternal overweight/obesity accounted for 1.1 million deaths in 1990, and 1.7 million deaths in 2010 [12, 13]. It is clear from the previous studies that maternal underweight, overweight or obesity are a threat to maternal and infant health [14–16]. For instance, high maternal BMI has closely related to gestational diabetes, gestational hypertension, cesarean section, preeclampsia, gestational age, preterm birth, fetal death, still birth, perinatal death, neonatal death, and infant death. The results are not consistent across studies. Some found higher risk and some lower risk of birth outcomes in connection to BMI. This discrepancy may be due small sample size, study design, regional variation, or country income categories [17].

Furthermore, like many developed countries, overweight and obesity are also increasing rapidly in Bangladesh. Despite this growing burden of BMI, very few studies have been conducted to date to examine the association between dual burden of maternal BMI and risk of adverse birth and health outcomes [18–20]. Most of these studies are limited to specific adverse outcomes and found mixed results between BMI and risk of maternal health outcomes. For instance, overweight and obesity was positively associated with risk of diabetes [21], and hypertension [22]. However, others found no significant association [23]. No study examined a comprehensive range of maternal health and birth outcomes in relation to maternal BMI using population based survey data. Thus, the present study is seeking to examine the association between maternal undernutrition and excessive body weight and risk of adverse birth and health outcomes.

## Methods

### Sources of data

**Fig. 1:**
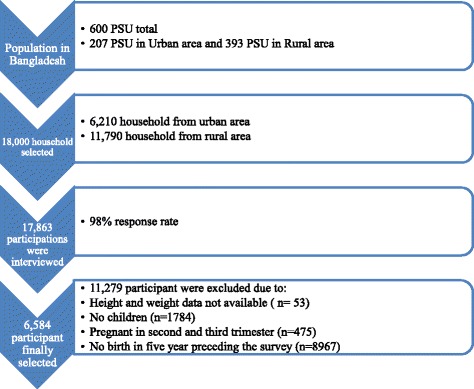
Sample selection

This study is based on a cross-sectional data from Bangladesh Demographic and Health survey (BDHS) – 2014. All information including socio-economic, demographic, anthropometric, birth and health characteristics were collected from 17,863 women aged 15 to 49 years. The key exclusion criteria were women having no children (*n =* 1,784), pregnant women in their second (13 weeks to 28 weeks of pregnancy) or third trimester (from 28 weeks of pregnancy to termination of pregnancy) (*n =* 475), and women who have not given birth in the last five years (*n =* 8,967). Non-response related to BMI (*n =* 53), pregnancy complications (*n =* 4), genital sore or ulcer (*n =* 9), occupation (*n =* 1) and number of antenatal visit (*n =* 5) were also excluded from the analysis. The final effective sample for analysis was 6,584. The participant’s selection framework is presented in Fig. 1. In addition, BDHS– 2011 data were extracted for diabetes, hypertension, preterm birth and prolonged labor.

### Exposure variable

The exposure variable in this study was maternal BMI. It was calculated by dividing weight in kilograms by the height in meters squared. The details measurements of women heights and weights were described in DHS website [24]. Using the World Health Organization (WHO) cut-off points, maternal BMI was categorized as:<18.5 kg/m^2^ (underweight), 18.5-24.9 kg/m^2^ (normal weight), 25–29.9 kg/m^2^ (overweight) and ≥ 30 kg/m^2^ (obese).

### Outcome variables

The study included a wide range of outcome variables including pregnancy complications (health problem during pregnancy that adversely affect mothers and fetus), pregnancy termination (termination of fetus before capable of independent life), genital sore/ulcer (bumps and lesions around the vagina), genital discharge (discharge thick, pasty, thin, cloudy, bloody liquid from the vagina), menstrual irregularities (menstrual cycle does not ranged between 21–35 days), cesarean delivery (surgical procedure of childbirth),diabetes (FBG ≥ 7.0 mmol/L) and hypertension (SBP ≥ 140 mmHg/ DBP ≥ 90 mmHg). Birth weight (babies born with weight <2500 g), stunting (child is shorter than for age), wasting (child weight is low as compared to height), early neonatal mortality (deaths at age 0–7 days), stillbirths (fetal death in pregnancies lasting seven or more months), perinatal mortality (fetal death after 7 months of pregnancy to first seven days of live born), preterm birth (<37 weeks of gestation) and prolonged labor (labor pain >12 h) were included as birth outcomes.

### Confounding adjustment

Different individual, household and community level characteristics were included in analysis as confounding adjustment variables. The variables were age (15–19, 20–24, 25–29, 30–34, 35–39, ≥40), respondents’ education (no formal education, primary, secondary, and higher education), spouse’s education, socio-economic status (poorest, poorer, average, richer, richest), region (southern, southeastern, central, western, mid-western, northwestern, eastern), place of residence (urban, rural), present working status (yes, no), and number of antenatal visits (no visit, less than or equal four visit, above four visit). We included diarrhea and acute respiratory infection in the adjusted models for stunting and wasting, as many previous studies found this variable significantly associated with child malnutrition.

### Statistical analysis

We used mean and frequency distributions to describe participant characteristics. We also estimated the prevalence of underweight and overweight by selected socio-demographic characteristics. Individual women were nested within household, households were nested within cluster/primary sampling unit, and clusters were nested within region. To account for multiple hierarchy and dependency in data, we performed multilevel (three levels) logistic regression models to examine the association between each outcome variable and BMI. Multilevel Poisson regression models were used when model convergence issues arose in the case of rare events. The initial model included only specific outcomes and BMI and the final model was adjusted for all potential confounding factors. All analyses accounted for probability sample design and we used Stata software version 13.1.

## Results

### Study characteristics

Table 1 summarizes the crude characteristics of the study subjects. We analyzed 6,584 married women who reported at least one birth within five years preceding the survey. On average, maternal age was 25.65 years. The crude mean BMI was 21.67 kg/m^2^ and systolic blood pressure was and 121.47 mmHg. The prevalence of diabetes and hypertension was 10% and 32% respectively. Around 47% maternal women reported pregnancy complication. Prevalence of low birth weight was 20%, cesarean delivery was 25% and pregnancy termination was 16%. Around 37% reported having a child with stunting and 14.4% reported a child with wasting.

**Table 1:** Study population characteristics

Characteristics	Crude prevalence
Mean (SE)	
Age (years)	25.65 (0.07)
Weight (kg)	54.74 (0.87)
Height (mm)	1563.37 (0.83)
Body mass index	21.67 (0.04)
Systolic blood pressure (mmHg)	121.47 (0.36)
Diastolic blood pressure (mmHg)	79.09 (0.19)
Percentage (95% CI)	
Underweight	21.9 (20.5-23.3)
Normal weight	58.9 (57.3-60.4)
Overweight	15.6 (14.3-17.0)
Obesity	3.9 (3.2-4.3)
Diabetes	9.9 (8.1-11.1)
Hypertension	31.7 (29.9-33.5)
Pregnancy complication	46.6 (44.1-49.1)
Genital sore	5.1 (4.4-5.9)
Genital discharge	10.7 (9.6-11.7)
Pregnancy termination	16.1 (15.0-17.4)
Menstrual irregularities	74.4 (72.9-75.9)
Low birth weight	20.0 (18.5-21.6)
Cesarean delivery	24.7 (22.6-27.0)
Stunting	36.5 (34.6-38.9)
Wasting	14.4 (13.4-15.6)
Preterm birth	31.3 (29.8-32.4)
Prolonged labor	29.2 (27.9-30.2)
Stillbirths	1.1 (0.8-1.2)
Early neonatal mortality	2.5 (2.1-3.0)
Perinatal mortality	2.4 (2.1-2.7)

SE, Standard error; 95% CI, 95% confidence interval

### Trend of underweight and overweight and obesity

**Fig. 2:**
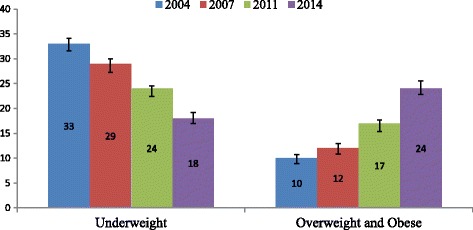
Trend in women’s nutritional status

Figure 2 presents the trends of underweight and overweight and obesity. The prevalence of underweight women decreased by around 15 percentage points (from 33% in 2004 to 18% in 2014). Additionally, prevalence of overweight and obese increased by 14 percentage points (from 10% in 2004 to 24% in 2014).

### Age-specific prevalence of BMI

**Fig. 3:**
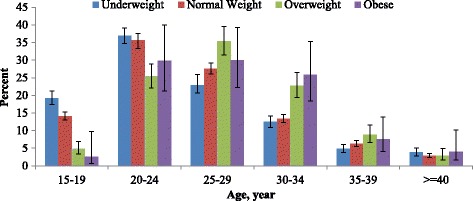
Age specific prevalence of BMI

Age-specific prevalence of underweight, overweight, and obesity is presented in Fig. 3. Significant variation observed in the prevalence of BMI across age categories. Higher prevalence of underweight (33.8%) and overweight (34.6%) was reported among women aged 20–24 years and 25–29 years, respectively.

### Socio-economic differentials of BMI

**Fig. 4:**
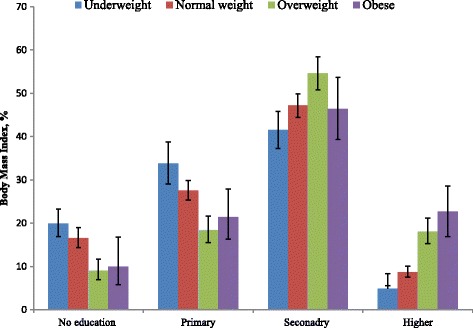
Prevalence of BMI by women education

Prevalence of BMI across women’s educational levels is presented in Fig. 4. The detailed prevalence of BMI according to the various demographic and socio economic characteristics is presented in Additional file: Table S1. Higher prevalence of underweight was found among women with primary (33.8%) and secondary education (41.5%). Overweight and obesity were considerably higher in women with secondary and higher education. So secondary school educated women were both at the higher risk of under- and over nutrition. Similar to women’s education level, women with higher educated husbands were also more likely to experience a higher prevalence of overweight and obesity. Noticeable regional variation of underweight and overweight prevalence was found in our study. More than one third of women in central city (Dhaka) were overweight and obese. Comparatively, a low prevalence of underweight women (6%) was observed in the Southern region (Barisal) (7%). In general, rural women were more likely to be underweight (82%) and urban women were more likely to be obese (48%). The result also shows that there was variation in underweight and overweight and obese among women across socio-economic status. Wealthy women were mainly overweight (40.8%) and obese (51.4%) and disadvantaged women were mainly underweight.

### Maternal BMI and risk of birth and health outcome

To assess the association between maternal BMI and birth and health outcomes, we performed a series of unadjusted and adjusted multilevel logistic models. The results of unadjusted and adjusted models for specific health and birth outcomes are shown in Tables 2 and 3. We performed likelihood test to choose preferable models. The tests compared random effects model against fixed effects model and found statistically significant results (*p* < 0.05). This implied the random effect models were necessary to perform clustering data.

**Table 2:** Maternal body mass index and risk of maternal health outcomes

	BMI group (kg/m^2^)	N (n)	Proportion (%)	OR (95% CI)	
				Unadjusted	Adjusted
Pregnancy complication	<18.5	752 (338)	45.0	1.03 (0.87-1.23)	1.07 (0.90-1.28)
	18.5-25	1986 (933)	47.0	1.00	1.00
	25-30	549 (280)	51.0	1.25 (1.02-1.53)	1.19 (0.96-1.46)
	≥30	136 (68)	50.0	2.23 (1.36-3.63)	2.05 (1.24-3.37)
Pregnancy Termination	<18.5	1471 (210)	14.3	0.88 (0.72-1.07)	0.91 (0.76-1.09)
	18.5-25	3782 (629)	16.6	1.00	1.00
	25-30	1055 (213)	20.2	1.38 (1.03-1.85)	1.14 (0.95-1.37)
	≥30	254 (54)	21.2	1.49 (1.02-2.25)	1.17 (0.84-1.62)
Genital sore/ulcer^a,b,c^	<18.5	1467 (91)	6.2	1.00 (0.98-1.02)	1.02 (0.69-1.52)
	18.5-25	3777 (222)	5.9	1.00	1.00
	≥25	1309 (72)	5.50	0.99 (0.98-1.01)	0.96 (0.63-1.36)
Genital discharge^a,b,c^	<18.5	1466 (213)	14.5	1.72 (1.06-2.77)	1.51 (0.94-2.42)
	18.5-25	3777 (421)	11.2	1.00	1.00
	≥25	1309 (98)	7.5	0.71 (0.53-0.93)	0.78 (0.53-1.13)
Menstrual irregularities^a^	<18.5	1467 (1052)	71.7	0.89 (0.78-1.03)	0.99 (0.86-1.14)
	18.5-25	3777 (2792)	73.9	1.00	1.00
	25-30	1055 (816)	77.3	1.19 (1.01-1.40)	0.98 (0.83-1.17)
	≥30	254 (200)	78.7	1.31 (1.02-1.81)	1.25 (0.76-2.06)
Cesarean delivery^a,d.e.f^	<18.5	1072 (144)	13.4	0.27 (0.11-0.67)	0.73 (0.52-1.03)
	18.5-25	2510 (581)	23.2	1.00	1.00
	25-30	608 (271)	34.6	6.67 (1.82-24.42)	1.67 (1.00-2.82)
	≥30	145 (76)	52.4	15.78 (2.27-109.57)	1.71 (1.02-3.31)
Diabetes^h^	18.5-25	3728 (349)	9.4	1.00	1.00
	25-30	196 (59)	30.1	4.04 (2.70-6.02)	2.58 (1.81-3.68)
	≥30	52 (10)	19.2	2.72 (1.21-6.14)	1.50 (0.56-2.36)
Hypertension^g^	18.5-25	3728 (1123)	30.1	1.00	1.00
	25-30	196 (117)	59.7	3.78 (2.74-5.22)	2.29 (1.60-3.29)
	≥30	52 (36)	69.2	6.60 (3.27-13.30)	4.12 (1.88-9.06)

N; Total sample and n; number of event; kg, Kilogram; m^2^, base unit of length

In some cases overweight and obesity were combined due to small sample size. The proportion (percent) and the result of multilevel logistic regression analysis were tabulated for each variable according to the BMI. All multilevel logistic regression analyses include age, respondent’s education, wealth index, region, place of residence and current working status. Additional confounding factors were included by the superscripts number (^a^ = children ever born, ^b^ = preceding birth interval, ^c^ = cesarean delivery, ^d^ = husband education,^e^ = household food security, ^f^ = antenatal mortality, ^g^ = diabetes, ^h^ = hypertension)

**Table 3:** Maternal BMI and adverse birth outcomes

	BMI group (kg/m^2^)	N (n)	Proportion (%)	ORorRR (95% CI)	
				Unadjusted	Adjusted
Stunting^a,b,c,d,e,h,i,m,n^	<18.5	1556 (706)	45.4	1.41 (1.25-1.59)	1.29 (1.11-1.40)
	18.5-25	4067 (1507)	37.1	1.00	1.00
	25-30	1097 (271)	24.7	0.55 (0.48-0.65)	0.80 (0.64-0.89)
	≥30	239 (59)	24.7	0.56 (0.41-0.75)	0.78 (0.49-1.20)
Wasting^a,b,c,d,e,h,i,m,n^	<18.5	1556 (312)	20.8	1.53 (1.31-1.78)	1.59 (1.13-1.80)
	18.5-25	4067 (572)	14.5	1.00	1.00
	25-30	1097 (100)	9.0	0.61 (0.49-0.76)	0.46 (0.34-0.86)
	≥30	239 (18)	4.5	0.50 (0.31-0.81)	0.49 (0.29-0.0.56)
Low birth weight^d,e,g,h^	<18.5	1072 (262)	24.4	0.62 (0.43-0.93)	0.73 (0.49-1.09)
	18.5-25	2507 (458)	18.3	1.00	1.00
	25-30	608 (101)	16.6	1.15 (0.85-1.55)	0.96 (0.72-1.29)
	≥30	144 (18)	12.5	1.72 (0.86-3.46)	1.36 (0.70-2.66)
Stillbirths^a,f,h*^	<18.5	1466 (22)	1.5	1.00 (0.99-1.01)	0.92 (0.52-1.63)
	18.5-25	3774 (54)	1.5	1.00	1.00
	25-30	1055 (10)	1.0	0.99 (0.98-1.00)	0.71 (0.32-1.58)
	≥30	253 (4)	1.58	1.00 (0.99-1.02)	3.20 (0.77-13.55)
Early neonatal mortality^a,f,h^	<18.5	637 (15)	2.4	0.99 (0.98-1.00)	0.87 (0.40-1.85)
	18.5-25	1797 (50)	2.9	1.00	1.00
	25-30	554 (11)	2.0	0.99 (0.98-1.01)	1.42 (0.50-4.07)
	≥30	119 (3)	2.5	1.00 (0.97-1.02)	1.08 (0.31-3.87)
Perinatal mortality ^a,f,h*^	<18.5	1466 (36)	2.5	1.00 (0.99-1.01)	0.87 (0.54-1.41)
	18.5-25	3774 (104)	2.8	1.00	1.00
	25-30	1055 (21)	2.0	0.99 (0.98-1.00)	1.21 (0.52-2.85)
	≥30	253 (7)	2.8	1.00 (0.97-1.02)	1.77 (0.64-4.89)
Prolonged labor^a,j,k,l*^	<18.5	75 (19)	25.3	0.98 (0.86-1.11)	1.90 (0.90-3.99)
	18.5-25	160 (44)	27.5	1.00	1.00
	≥25	23 (10)	43.5	1.17 (0.96-1.43)	7.57 (1.26-45.43)
Preterm birth^a,j,k,l*^	<18.5	75 (18)	24.0	0.88 (0.77-1.00)	0.43 (0.20-0.92)
	18.5-25	161 (59)	16.8	1.00	1.00
	≥25	24 (5)	20.8	0.85 (0.70-1.04)	0.28 (0.08-0.94)

N; Total sample and n; number of event; kg, Kilogram; m^2^, base unit of length

In some cases overweight and obesity were combined due to small sample size. The proportion (percent) and the result of three level logistic regression analysis (*multilevel Poisson regression) were tabulated for each variable according to the BMI. All multilevel regression analysis includes age, respondent’s education, wealth index, region, place of residence. Additional confounding factors are included by the superscripts number (^a^ = body mass index, ^b^ = age of child, ^c^ = birth order, ^d^ = husband education, ^e^ = household food security, ^f^ = current working status, ^g^ = children ever boron, ^h^ = antenatal care, ^i^ = low birth weight, ^j^ = anemia, ^k^ = gestational diabetes, ^l^ = gestational hypertension, ^m^ = diarrhea, ^n^ = acute respiratory infection)

The results indicated that pregnancy complications were higher among obese (adjusted odds ratio (AOR), 2.05; 95% CI, 1.24-3.37) women than normal-weight women. The risk of pregnancy termination was relatively higher in overweight and obese women than the normal weight women; however, the association was statistically insignificant. We did not find any significant association between maternal BMI and risk of genital sore/ulcer, genital discharge, menstrual irregularities, and low birth weight. Both unadjusted and adjusted models showed that maternal BMI was positively associated with diabetes, hypertension, and cesarean delivery.

Overweight and obese women had 1.67 times (95% CI, 1.00-2.82) and 1.71 times (95% CI, 1.02-3.31) higher risk, respectively, for cesarean delivery as compared to normal-weight women. The fully adjusted model showed that overweight women were 2.58 times (95% CI, 1.81-3.68) more likely of developing diabetes than normal-weight women. As compared to normal-weight women, overweight and obese women were 2.29 times (95% CI, 1.60-3.29) and 4.12 times (95% CI, 1.88-9.06) higher risk of developing hypertension, respectively.

The multilevel logistic regression results indicated that the risk of stunting and wasting was 1.29 times (95% CI, 1.11-1.40) and 1.59 times (95% CI, 1.13-1.80) higher, respectively, among underweight women than normal weight women. However, overweight and obese women played a protective role against stunting and wasting. Both underweight and overweight or obese women were lower risk of preterm birth compared to normal weight women. Compared to normal weight women, the risk of prolong delivery was higher among overweight and obese (adjusted risk ratio (ARR), 7.57; 95% CI, 1.26-45.43) women. Overweight and obese women were higher risk of stillbirths (ARR, 3.20; 95% CI, 0.77-13.55), early neonatal mortality (ARR, 1.42; 95% CI, 0.50-4.07) and perinatal mortality (ARR, 1.77; 95% CI, 0.64-4.89). However, these increased risks were not statistically significant.

## Discussion

This is the first nationally representative cross-sectional study in Bangladesh that has demonstrated the risk of birth and health outcomes in connection to maternal BMI. The findings suggest that between 2004 and 2014, maternal overweight and obesity increased to 240% in Bangladesh, while undernutrition declined to 54%. Maternal overweight or obese increased risk of pregnancy complication, cesarean delivery, diabetes, and hypertension and prolonged labor. The study also found that the risk of stunting and wasting was higher among underweight women than normal weight women.

In general, over time the prevalence of overweight and obesity is increasing and underweight is decreasing. In 2014, prevalence of underweight and overweight was 21.9% and 15.6%, respectively. This situation represents a double burden of nutrition condition that exists among women in Bangladesh. Similar nutritional problems are also observed in India [25], and others developing countries [26–28]. There was an apparent socio-economic, demographic and regional distribution of underweight and overweight or obese. Women in low socio-economic status, with little or no education and rural residence experienced greater risk of underweight. Conversely, higher educated women, wealthier women and those residing in urban areas had higher prevalence of overweight and obesity. This is consistent with previous studies from many settings including Bangladesh [29], and others South Asian countries [25, 30].

Over the last few decades, Bangladesh has substantially reduced maternal mortality [31, 32]. In addition, infant and neonatal mortality, and total fertility rates have also declined sharply since 1990. An estimated 11,000-21,000 mothers die each year in Bangladesh due to the pregnancy related complication [33], and a further 320,000 women suffer from the injuries or disabilities caused by these complication during pregnancy and child birth [34]. Available findings suggest that controlling weight may significantly reduce the number of pregnancy complication. Thus individual and national level awareness about such adverse effect of overweight and obesity may lead to reduce the number of pregnancy complication which ultimately crop the frequency of mothers death and disabilities.

The association between diabetes and overweight (or obesity) in general population is well established in Bangladesh [35, 36], and other countries [37, 38]. However, comprehensive assessment especially among women is still lacking in Bangladesh. Study results indicate that overweight and obesity have an increased risk of developing diabetes among women in Bangladesh. Additionally this study found a substantially higher proportion of hypertension among overweight and obese women, a finding supported by others studies [39, 40]. A recent meta-analysis based on low- and lower middle-income countries also found the positive association between the maternal overweight and obesity with gestational diabetes, gestational hypertension, preeclampsia and post partum haemarroage [17]. All of these non-communicable diseases have become leading risk factors for deaths and disability in developing and developed countries [12]. In 2010, hypertension accounted for 2.9 million deaths among women (6% of total Disability Adjusted Life Years (DALYs)), high fasting plasma glucose accounted for 1.1 million deaths (3% of DALYs), ischemic heart diseases accounted for 1.7 million deaths (3% of DALYs), and stroke accounted for 2.0 million deaths (4% of DALYs) in developing countries [13].

Consistent with previous studies [41, 42], overweight and obese women were more likely to have cesarean delivery in our study. Weight management is important for every woman in reproductive age. Women with a normal BMI should strive for maintenance of a healthy weight, whereas underweight and overweight women should aim to healthy weight prior to the pregnancy. These contribute to reduce the effect of high maternal BMI on labor and delivery complications and cesarean delivery [42].

On average, the study found 15 stillbirths and 37 early neonatal deaths per 1000 live births. This is consistent with other low- and lower middle-income countries [43, 44]. Deaths during the neonatal period contribute around 38% of the total under five deaths [44]. The main causes of neonatal death are preterm birth (28%), severe infection (26%) and low birth weight (31%) [44, 45]. Complication during pregnancy and delivery is also another important determinant of neonatal survival [46]. The risk of overweight and obesity on these adverse outcomes is well known which greatly contribute to the increased risk of stillbirth, early neonatal mortality and perinatal mortality. Consistent with previous literature [47, 48], obese women were more likely to experience stillbirth, early neonatal mortality and perinatal mortality. Thus, it is necessary to emphasis-that women control their weight and reduce levels of overweight or obesity to reduce these adverse birth outcomes.

Similar to some previous studies [49, 50], overweight and obese women were more likely to have prolonged labor, which may lead to maternal and newborn death and disability [51]. It may also result in increased risk of cesarean delivery and maternal complication following ruptured membranes, trauma to the bladder, and ruptured uterus with consequent haemorrhage [52]. In this study, both underweight and overweight or obese women were less likely to experience preterm birth compared to normal-weight women. This is inconsistent with other studies conducted in Ireland [53], Spain [42], South Australia [54], and China [55]. A recent systematic review and meta-analysis also found underweight women have higher risk of preterm birth [17]. This contradictory finding in our study may be due to the small number of cases was extracted from the verbal autopsy record. Our study indicated that underweight women were at increased risk of stunting and wasting children. However, overweight or obesity plays a protective role against stunting and wasting among children

The key strength of this study was the availability of a large, nationally representative dataset providing the sufficient power to investigate the adverse birth and health outcome related to maternal BMI. The use of STROBE checklist strengthened our paper (Additional file 1: Table S2). However, we were unable to establish a causal relationship between the maternal BMI and birth and health outcomes as a result of cross sectional study design. Secondly, BMI should be measured either before pregnancy or in the first trimester; however, BDHS collected women height and weight information during interview and birth outcomes were recorded within last three years. Since BMI is not stable over time, which may lead to partially bias the association between exposure and outcomes. To address this limitation, we exclude women with substantial fluctuation of BMI. Furthermore, we see reassuringly that the direction and magnitude of effect of BMI as a function of time elapsed between the index of birth and survey is similar in women with adverse birth or health outcomes, and would if anything have led to underestimate of effect. These findings suggest that misclassification bias of BMI is likely to be small. A previous study published in *Lancet* also found the little misclassification bias of BMI in a similar study design [56]. Finally, information about a majority of the outcome variables (except diabetes and hypertension) were self-reported. However, the DHS has been collecting data in low-income settings by similar methods for more than two decades, and there has been a substantial improvement in the completeness and reliability of the dataset [57].

## Conclusions

Our findings represent a clear picture of the adverse birth and health outcome that are related to maternal BMI. Higher risk of pregnancy complication, prolonged labor, cesarean delivery, diabetes, and hypertension was found among overweight or obese women. On the other hand, higher odds of stunting, and wasting were found among underweight women. Therefore, weight management is necessary to prevent adverse birth and health outcomes. Clinicians and other healthcare providers and policy maker should counsel women prior to or in early pregnancy on adverse threats of underweight, overweight, or obesity on their own and their infant’s health. Informed women could try to optimize their BMI before conception.

## Additional file

Additional file 1: Table S1. Percentage distribution of the respondents by some selected socio economic characteristics and BMI. **Table S2.** STROBE Statement—checklist of items that should be included in reports of observational studies. (DOC 134 kb)

## Abbreviations

BMI: Body mass index
DHS: Demographic and health survey
OR: Odds ratio
RR: Relative risk
