# Supplementary material for: Maternal undernutrition and excessive body weight and risk of birth and health outcomes
Source: Arch Public Health. 2017 Feb 3;75:12. doi: 10.1186/s13690-017-0181-0 (PMC5291969; doi:10.1186/s13690-017-0181-0)
Supplement: Additional file 1: Table S1. — Percentage distribution of the respondents by some selected socio economic characteristics and BMI. Table S2. STROBE Statement—checklist of items that should be included in reports of observational studies. (DOC 134 kb) [file 13690_2017_181_MOESM1_ESM.doc]

Additional file 1: Table S1. Percentage distribution of the respondents by some selected socio economic characteristics and BMI

| Characteristics | | | | | Total (n) | Body Mass Index, percent (95% CI) | | | |  |
| --- | --- | --- | --- | --- | --- | --- | --- | --- | --- | --- |
|  |  |  |  |  |  | Underweight | Normal weight | Overweight | Obese | P values |
| Respondents age (In years) | | | | | |  |  |  |  |  |
| 15-19 | 1010 (15.4) | | | | | 24.7 (22.3-27.2) | 15.3 (13.9-16.7)) | 6.4 (4.9-8.1) | 4.9 (2.6-9.1) | <0.001 |
| 20-24 | 2111 (32.2) | | | | | 33.8 (30.4-37.3) | 33.5 (31.4-35.6) | 26.8 (23.7-30.1) | 22.7 (17.1-29.5) |  |
| 25-29 | 1802 (27.5) | | | | | 22.4 (19.9-24.9) | 27.8 (26.1-29.7) | 34.6 (31.0-38.3) | 28.1 (21.9-35.3) |  |
| 30-34 | 1055 (16.1) | | | | | 13.1 (10.9-15.5) | 15.4 (13.8-17.2) | 22.3 (19.3-25.7) | 28.0 (21.8-35.3) |  |
| 35-39 | 434 (6.6) | | | | | 4.4 (3.4-5.7) | 6.1 (5.2-7.2) | 8.2 (6.5-10.3) | 13.4 (9.3-18.9) |  |
| 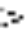40 | 136 (2.1) | | | | | 1.7 (1.1-2.7) | 1.9 (1.4-2.5) | 1.8 (1.1-2.9) | 2.8 (1.1-6.8) |  |
| Respondents education | | | | | |  |  |  |  |  |
| None | | 988 (15.1) | | | | 19.9 (16.9-23.3) | 16.5 (14.3-19.0) | 9.0 (6.9-11.7) | 9.9 (5.8-16.8) | <0.001 |
| Primary^1^ | | 1763 (26.9) | | | | 33.8 (29.1-38.8) | 27.6 (25.3-29.9) | 18.4 (15.5-21.6) | 21.4 (16.3-27.9) |  |
| Secondary^2^ | | 3066 (46.8) | | | | 41.5 (37.3-45.8) | 47.2 (44.5-49.9) | 54.6 (50.8-58.4) | 46.4 (39.3-53.7) |  |
| Higher | | 731 (11.2) | | | | 4.9 (3.7-6.4) | 8.7 (7.5-10.1) | 18.1 (15.3-21.2) | 22.7 (16.9-28.6) |  |
| Religion | |  | | | |  |  |  |  |  |
| Islam | | 5995 (91.6) | | | | 92.5 (89.5-94.7)) | 91.1 (87.9-93.4) | 90.7 (86.6-93.6) | 88.9 (81.1-93.8) | 0.67 |
| Others | | 552 (8.4) | | | | 7.5 (5.3-10.5) | 8.8 (6.6-11.7) | 9.3 (6.4-13.4) | 11.3 (6.2-18.9) |  |
| Working status | | | | | |  |  |  |  |  |
| No | | 4872 (74.4) | | | | 74.9 (71.6-78.0) | 71.3 (68.8-73.6) | 74.8 (71.1-78.2) | 87.2 (82.1-91.0) | <0.001 |
| Yes | | 1674 (25.6) | | | | 25.0 (21.9-28.4) | 28.7 (26.4-31.2) | 25.2 (21.8-28.9) | 12.8 (8.9-17.9) |  |
| Husband education | | | | | |  |  |  |  |  |
| None | | 1588 (24.3) | | | | 33.6 (29.9-37.5) | 25.7 (23.4-28.2) | 12.9 (10.7-15.6) | 25.0 (22.9-27.3) | <0.001 |
| Primary^1^ | | 1944 (29.6) | | | | 34.4 (31.7-37.3) | 30.3 (28.4-32.3) | 22.3 (19.3-25.6) | 29.6 (28.1-31.2) |  |
| Secondary^2^ | | 2024 (30.9)) | | | | 25.3 (21.9-28.9) | 31.7 (29.5-34.1) | 37.7 (33.9-41.7) | 31.2 (29.3-33.2) |  |
| Higher | | 991 (15.1) | | | | 6.7 (5.5-8.3) | 12.2 (10.8-13.7) | 30.7 (24.2-38.1) | 14.1 (12.8-15.5) |  |
| Husband occupation | | | | | |  |  |  |  |  |
| Agriculture | | | | 1577 (24.2) | | 31.5 (27.5-35.8) | 27.2 (24.5-30.1) | 14.5 (11.6-17.8) | 11.4 (7.2-17.6) | <0.001 |
| Physical | | | | 2877 (44.1) | | 46.7 (43.2-50.2) | 44.4 (41.8-47.1) | 39.3 (35.4-43.3) | 42.1 (34.4-50.2) |  |
| Services | | | | 440 (6.7) | | 2.9 (2.0-4.1) | 4.9 (4.1-5.9) | 11.1 (8.8-13.8) | 17.4 (11.9-24.9) |  |
| Business | | | | 1471 (22.6)) | | 16.2 (13.9-18.9) | 20.9 (19.2-22.8) | 32.8 (28.9-36.9) | 27.8 (21.8-34.7) |  |
| Others | | | | 159 (2.4) | | 2.8 (1.9-4.1) | 2.6 (1.9-3.5) | 2.4 (1.5-3.9) | 1.2 (0.5-3.3) |  |
| Region | | | |  | |  |  |  |  |  |
| Southern | | | | 792 (12.1) | | 6.9 (5.6-8.7) | 5.9 (5.0-6.8) | 5.6 (3.9-8.3) | 3.4 (2.2-5.3) | <0.001 |
| Southeastern | | | | 1222 (18.7) | | 19.0 (16.1-22.2) | 20.4 (17.9-23.1) | 25.3 (21.8-29.1) | 23.2 (17.4-30.3) |  |
| Central | | | | 1181 (18.0) | | 32.7 (27.5-38.4) | 36.2 (31.6-41.1) | 38.2 (33.6-43.1) | 41.3 (34.5-48.5) |  |
| Western | | | | 752 (11.5) | | 6.6 (5.6-7.8) | 8.2 (7.1-9.3) | 8.8 (7.0-11.0) | 8.1 (5.5-11.6) |  |
| Mid-western | | | | 824 (12.6) | | 12.4 (10.0-15.3) | 10.2 (8.9-11.6) | 9.0 (7.4-10.9) | 10.8 (7.5-15.2)) |  |
| Northwestern | | | | 861 (13.2) | | 10.5 (8.8-12.4) | 11.3 (9.5-13.4) | 7.9 (5.9-10.9) | 6.4 (4.1-9.7) |  |
| Eastern | | | | 916 (14.0) | | 11.9 (9.9-14.3) | 7.9 (6.0-10.4) | 6.4 (4.1-9.7) | 6.9 (4.5-10.4) |  |
| Place of residence | | | | | |  |  |  |  |  |
| Urban | | | 2137 (32.6) | | | 18.2 (15.1-21.8) | 24.1 (21.7-26.8) | 41.9 (37.5-46.5) | 48.1 (41.2-54.9) | <0.001 |
| Rural | | | 4411 (67.4) | | | 81.8 (78.2-84.9) | 75.9 (73.2-78.4) | 58.1 (53.5-62.5) | 51.9 (45.2-54.7) |  |
| Wealth Index^2^ | | |  | | |  |  |  |  |  |
| Poorest | | | 1344 (20.5) | | | 31.8 (28.0-35.8) | 21.6 (18.9-24.4) | 6.3 (4.6-8.6) | 7.6 (4.1-13.6) | <0.001 |
| Poorer | | | 1225 (18.7) | | | 25.3 (22.5-28.2) | 19.3 (17.5-21.2) | 10.2 (6.3-12.6) | 6.8 (3.8-11.7) |  |
| Middle | | | 1286 (19.6) | | | 19.4 (16.7-22.3) | 20.6 (18.0-23.4) | 19.3 (15.9-23.3) | 12.4 (8.3-18.2) |  |
| Richer | | | 1357 (20.7) | | | 15.6 (12.6-19.2) | 21.5 (19.3-23.8) | 23.3 (19.7-27.4) | 21.8 (16.1-29.1) |  |
| Richest | | | 1336 (20.4) | | | 8.0 (6.1-10.4) | 17.1 (14.8-19.7) | 40.8 (36.3-45.4) | 51.4 (43.8-58.9) |  |
| Total | | | 6584(100.0) | | | 21.9 (20.5-23.3) | 58.9 (57.3-60.4) | 15.6 (14.3-16.9) | 3.7 (3.2-4.3) |  |

Note: The numbers inside the parentheses represent the percentages, ^1^Primary completed is defined as completing grade 5, ^2^ Secondary completed is defined as completing grade 10, ^2^Wealth index measure the socio economic status

Table S2. STROBE Statement—checklist of items that should be included in reports of observational studies

|  | Item No | Recommendation | Page/line # |
| --- | --- | --- | --- |
| **Title and abstract** | 1 | (*a*) Indicate the study’s design with a commonly used term in the title or the abstract | Pg 3, line 4 |
|  |  | (*b*) Provide in the abstract an informative and balanced summary of what was done and what was found | Pg 3, line 11-18 |
| Introduction | | |  |
| Background/rationale | 2 | Explain the scientific background and rationale for the investigation being reported | Pg 4-5, line 26-53 |
| Objectives | 3 | State specific objectives, including any prespecified hypotheses | Pg5, line 53-60 |
| Methods | | |  |
| Study design | 4 | Present key elements of study design early in the paper | Pg5, line64; |
| Setting | 5 | Describe the setting, locations, and relevant dates, including periods of recruitment, exposure, follow-up, and data collection | Pg5, line64 |
| Participants | 6 | (*a*) *Cross-sectional study*—Give the eligibility criteria, and the sources and methods of selection of participants | Pg 5-6, line 64-72 |
|  |  |  |  |
| Variables | 7 | Clearly define all outcomes, exposures, predictors, potential confounders, and effect modifiers. Give diagnostic criteria, if applicable | Pg 6, line73-101 |
| Data sources/ measurement | 8* | For each variable of interest, give sources of data and details of methods of assessment (measurement). Describe comparability of assessment methods if there is more than one group | NA |
| Bias | 9 | Describe any efforts to address potential sources of bias | NA |
| Study size | 10 | Explain how the study size was arrived at |  |
| Quantitative variables | 11 | Explain how quantitative variables were handled in the analyses. If applicable, describe which groupings were chosen and why | Pg6-7, line 94-101 |
| Statistical methods | 12 | (*a*) Describe all statistical methods, including those used to control for confounding | Pg7, line 103-113 |
|  |  | (*b*) Describe any methods used to examine subgroups and interactions | NA |
|  |  | (*c*) Explain how missing data were addressed |  |
|  |  | (*d*) *Cross-sectional study*—If applicable, describe analytical methods taking account of sampling strategy | Pg7, line 112-113 |
|  |  | (*e*) Describe any sensitivity analyses | NA |

Continued on next page

| Results | | | Page/line # |
| --- | --- | --- | --- |
| Participants | 13* | (a) Report numbers of individuals at each stage of study—eg numbers potentially eligible, examined for eligibility, confirmed eligible, included in the study, completing follow-up, and analysed | Pg5-6, line 65-72; Fig 1 |
|  |  | (b) Give reasons for non-participation at each stage |  |
|  |  | (c) Consider use of a flow diagram |  |
| Descriptive data | 14* | (a) Give characteristics of study participants (eg demographic, clinical, social) and information on exposures and potential confounders | Pg8; line115-22; Fig 2,3,4 |
|  |  | (b) Indicate number of participants with missing data for each variable of interest |  |
|  |  |  |  |
| Outcome data | 15* | *Cross-sectional study—*Report numbers of outcome events or summary measures | Table 2 Table *3* |
| Main results | 16 | (*a*) Give unadjusted estimates and, if applicable, confounder-adjusted estimates and their precision (eg, 95% confidence interval). Make clear which confounders were adjusted for and why they were included | Pg 9-10, line150-79 |
|  |  | (*b*) Report category boundaries when continuous variables were categorized |  |
|  |  | (*c*) If relevant, consider translating estimates of relative risk into absolute risk for a meaningful time period |  |
| Other analyses | 17 | Report other analyses done—eg analyses of subgroups and interactions, and sensitivity analyses | NA |
| Discussion | | |  |
| Key results | 18 | Summarise key results with reference to study objectives | Pg11-14 line 181-249 |
| Limitations | 19 | Discuss limitations of the study, taking into account sources of potential bias or imprecision. Discuss both direction and magnitude of any potential bias | Pg13-14, line 250-61 |
| Interpretation | 20 | Give a cautious overall interpretation of results considering objectives, limitations, multiplicity of analyses, results from similar studies, and other relevant evidence |  |
| Generalisability | 21 | Discuss the generalisability (external validity) of the study results | Pg13, line 250-52 |
| Other information | | |  |
| Funding | 22 | Give the source of funding and the role of the funders for the present study and, if applicable, for the original study on which the present article is based | Pg15, line288-89 |

*Give information separately for cases and controls in case-control studies and, if applicable, for exposed and unexposed groups in cohort and cross-sectional studies.
